# Microwave-induced fast crystallization of amorphous hierarchical anatase microspheres

**DOI:** 10.1186/1556-276X-9-273

**Published:** 2014-05-29

**Authors:** David G Calatayud, Teresa Jardiel, Marco Peiteado, Amador C Caballero, Daniel Fernández-Hevia

**Affiliations:** 1 Instituto de Cerámica y Vidrio, CSIC, c/Kelsen 5, Campus de Cantoblanco, Madrid 28049, Spain; 2 POEMMA-CEMDATIC, ETSI Telecomunicación, (UPM), Av. Complutense 30, Madrid 28040, Spain; 3 Department of Chemistry, Group of Photocatalysis and Spectroscopy Applied to the Environment (FEAM), Universidad de Las Palmas de Gran Canaria, Campus de Tafira, Gran Canaria 35017, Spain; 4 INAEL Electrical Systems, S.A. c/Jarama 5, Toledo 45007, Spain

**Keywords:** TiO2 anatase, Microwave heating, Nanoparticles, Hierarchical microspheres, Photocatalysis

## Abstract

The fabrication of hierarchical anatase microspheres with potential photocatalytic properties eventually comprises a consolidation step in which a high degree of crystalline order is typically achieved through conventional electric heating treatments. This however entails a substantial reduction in the specific surface area and porosity of the powders, with the consequent deterioration in their photocatalytic response. Here, we have tested the employ of microwave heating as an alternative energy-saving sintering method to promote fast crystallization. The results obtained suggest that under the microwave radiation, the TiO_2_ hierarchical structures can effectively crystallize in a drastically reduced heating time, allowing the specific surface area and the porosity to be kept in the high values required for an improved photocatalytic performance.

## Background

The unique physicochemical properties of TiO_2_ nanoparticles have lately attracted a tremendous interest in a wide range of scientific and technological fields [1-5]. Of particular interest for its potential photocatalytic applications to environmental purification, hydrogen generation and/or solar energy conversion is the preparation of hierarchical structures in which TiO_2_ anatase nanoparticles are assembled into organized configurations at a microscopic level [6-11]. On one hand, hierarchical structures may attain low density, high crystallinity and a large specific surface area, structural parameters all required to improve the photocatalytic performance. On the other hand, the micrometric size of the organized assemblies will allow an easy recovery of the photocatalyst from the working suspension after use. In this context, different synthesis strategies have been recently tested to prepare TiO_2_ hierarchical structures. For example, using templates and/or applying hydro(solvo)thermal conditions, anatase nanostructures assembled onto micron-sized spherical units have been synthesized initially showing a high stability and a monodisperse nature that can satisfy the abovementioned characteristics [12-15]. The main problem with all these methods is the subsequent thermal treatment at mild/high temperatures, which, being necessary to increase the crystallinity of the samples, also reduces their porosity and specific surface area. Eventually, this provokes a severe devaluation of their photocatalytic performance which hampers the practical application of these powders. Bearing this in mind, in this contribution, we propose to replace the conventional thermal treatment by a microwave heating process, an alternative and energy-saving sintering technique which has been successfully employed for the consolidation of some ceramic systems [16-19]. Microwave radiation may induce a fast crystallization of the amorphous hierarchical anatase microspheres, simultaneously keeping the constituent nanoparticles with a high specific surface area and a high porosity level necessary for a good photocatalytic activity.

## Methods

The chemicals titanium (IV) tetrabutoxide (Ti(OBut)_4_, 98%, Fluka, St. Louis, MO, USA) and anhydrous ethanol (EtOH, analytically pure, Merck, Whitehouse Station, NJ, USA) were used without further purification. TiO_2_ microspheres have been obtained following a facile methodology previously developed by our group [20]. In essence, a solution of Ti(OBut)_4_ in 1 L of absolute ethanol is stirred at room temperature, and after 6.5 h, it is evaporated to dryness under atmospheric conditions. The evolved white precipitate is washed with water and ethanol thoroughly and dried at room temperature. The obtained powder is spread on a high-density alumina crucible placed on the top of a microwave susceptor element, and microwave heating is finally applied at 700 W for different time intervals using a commercial Tesco microwave oven (Chestnut, England, UK). For comparison, a small fraction of the as-precipitated powder is subjected to a conventional heating at 400°C/1 h on electric furnace.

The analyses of the crystalline structure and the phase identification were performed by X-ray diffraction (XRD Bruker D8 ADVANCE, Madison, WI, USA) with a monochromatized source of Cu-Kα_1_ radiation (*λ* = 1.5406 nm) at 1.6 kW (40 KV, 40 mA); samples were prepared by placing a drop of a concentrated ethanol dispersion of particles onto a single crystal silicon plate. Powder samples were initially characterized using a Hitachi TM1000 tabletop scanning electron microscope (Chiyoda-ku, Japan) working on backscattered mode. Field-emission scanning electron microscopy (FESEM) images were obtained with a Hitachi S-4700 working at 20 kV. The specific surface area was determined by the Brunauer-Emmett-Telle (BET) method in a Monosorb Analyzer MS-13 QuantaChrome (Boca Raton, FL, USA). Nitrogen adsorption/desorption isotherms were carried out on an ASAP 2020-Micromeritics (Norcross, GA, USA) at 77 K. Samples were degassed at 30°C during 48 h before analysis. Transmission electron microscopy (TEM) images were obtained on a JEOL 2100 F TEM/STEM (Tokyo, Japan) operating at 200 kV and equipped with a field emission electron gun providing a point resolution of 0.19 nm; samples were prepared by placing a drop of a dilute ethanol dispersion of nanoparticles onto a 300-mesh carbon-coated copper grid and evaporated immediately at 60°C.

### Testing of photocatalytic activity

The photocatalytic performance of the powders prepared in this study was evaluated in the following way: 50 mg of powder were initially suspended in an aqueous solution of methyl orange (10^-5^ M, 100 mL) using a quartz reactor. The suspension, kept under magnetic stirring, was then irradiated using a high-pressure mercury vapour lamp (250 W, HPL-N Philips, Amsterdam, The Netherlands) and 4 ml aliquots were taken progressively from the suspension after different irradiation times. The supernatant and the solid particles were separated by centrifugation at 6,000 rpm. The absorption spectrum of the supernatant solution was measured on a Perkin Elmer Lambda 950 UV/vis spectrometer (Waltham, MA, USA), and the concentration (degradation) of methyl orange was determined monitoring the changes in the absorbance at 465 nm. On collecting these data, two side effects must be considered which may lead to a misinterpreted decreased value in the methyl orange concentration: the self-degradation of the methyl orange molecule under the irradiation, as well as its incidental (partial) absorption to the surface of the TiO_2_ particles. In this contribution, both scenarios were contemplated as follows: on one hand, a blank solution of methyl orange with no TiO_2_ powder was irradiated under the same experimental conditions; as it was observed, in the absence of our anatase particles, no degradation of methyl orange was indeed produced. On the other hand, suspensions with methyl orange and the different TiO_2_ powders were prepared as described before but they were not subjected to irradiation: in such dark conditions, no changes in the methyl orange concentration were observed for these suspensions all along the test, so absorption to the TiO_2_ surface was discarded in all cases.

## Results and discussion

**Figure 1:**
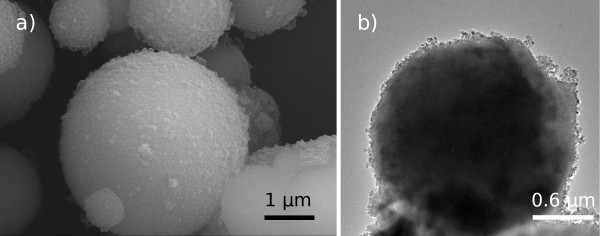
**FESEM (a) and TEM (b) micrographs of the Ti**_**sph **_**as-prepared powder.**

FESEM and TEM micrographs in Figure 1 shows the TiO_2_ powder as synthesized following the methodology described. It is constituted by spherical particles with a mean size around 1 to 2 μm and formed in turn by the agglomeration of a myriad of smaller nanoparticles. Furthermore, this hierarchical configuration, from now labelled as Ti_sph_ powder, displays an outstanding specific surface area, as large as *S*_s_ = 322 m^2^ · g^-1^, indicating the presence of interparticle porosity (meso- and microporosity).

**Figure 2:**
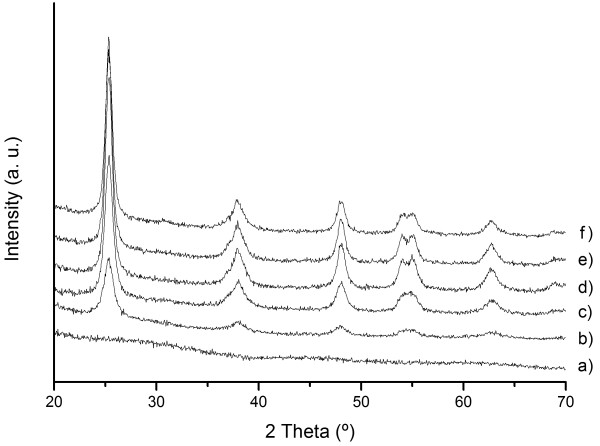
**X-ray diffractograms.** Of as-synthesized Ti_sph_ powder (curve a) and after 7 min (curve b), 10 min (curve c), 15 min (curve d) and 30 min (curve e) of MW treatment. XRD of the same powder treated at 400°C/1 h in a conventional electric furnace (f). All peaks corresponding to TiO_2_ anatase (JCPDS file no. 21-1272).

Certainly, such a high specific surface on a micron-sized powder may have tremendous potential for photocatalytic applications, but when going to XRD measurements, no trace of crystalline order was ever observed, see Figure 2a. This represents a serious problem since as mentioned, a high degree of crystallinity is essential for an efficient photocatalytic performance. In fact, photoreactivity demands a compromise between crystallinity, specific surface and porosity, so here is where we took our amorphous Ti_sph_ powder to fast microwave crystallization, trying to improve the crystallinity of the TiO_2_ spheres with the minor loss in specific surface area and porosity (i.e. keeping the hierarchical microstructure). In this sense, Figure 2 evinces that after 7 min of microwave (MW) radiation, XRD peaks of the TiO_2_ anatase phase can be already detected in the powder sample. As the exposure time is increased, an increase in the structural order is also observed (narrower peaks) and after 15 min, no further improvement in the crystallinity seems to be attained with the MW treatment. Moreover, the XRD analyses also showed that 10 min under MW radiation produced a crystallinity comparable to that obtained after 1 h at 400°C in a conventional electric furnace (similar width of XRD peaks in diffractograms of Figure 2c and 2f).

**Figure 3:**
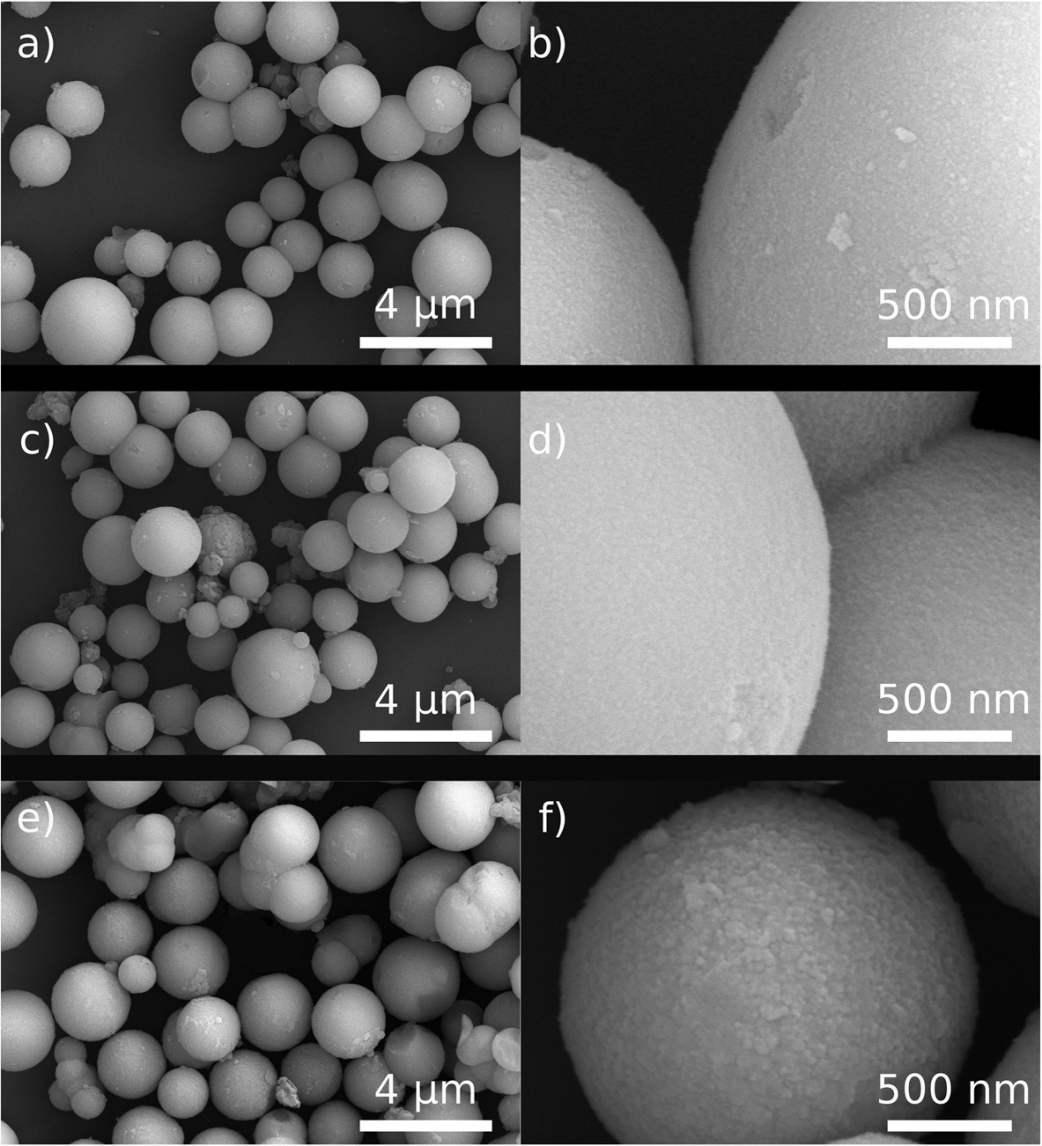
**FESEM micrographs of the Ti**_**sph **_**powder.** After being exposed to different thermal treatments, 7 min under MW radiation **(a, b)**, 15 min under MW radiation **(c, d)** and 1 h of conventional electric heating at 400°C **(e, f)**.

When going to the microscope, Figure 3 shows that the spherical morphology is retained after all the heating treatments. Also, no changes have been produced in the size of the TiO_2_ spheres, still composed by the assembly of innumerable nanoparticles. This nanostructure was investigated by specific surface area measurements, and as inferred from the data summarized in Table 1, the decrease in the specific surface area is less pronounced for the sample exposed 10 min to the microwaves (113 m^2^/g) than for the powder conventionally heated in the electric furnace (82 m^2^/g), although both powders exhibit a similar crystallinity by XRD.

**Table 1:** Specific surface area of the prepared samples

**Sample**	**Specific surface area (±1 m**^**2**^**/g)**
As-synthesized Ti_sph_ powder	322
7 min MW heating	232
10 min MW heating	113
15 min MW heating	75
30 min MW heating	65
400°C/1 h conventional heating	82

**Figure 4:**
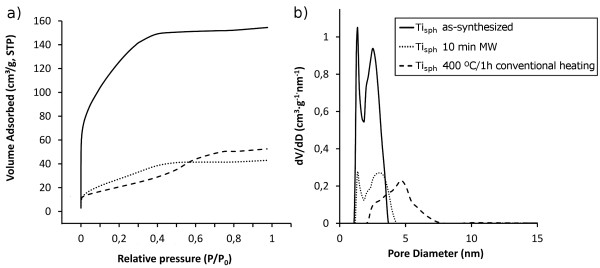
Nitrogen adsorption-desorption BET isotherms (a) and pore size distribution curves (b).

In addition, the pore structure of the samples was analyzed by N_2_ adsorption/desorption measurements, the pore size distribution being calculated by the density functional theory method. The BET isotherms in Figure 4a are in agreement with the observed decrease in the specific surface area after the thermal treatments. Regarding the pore size, a bimodal distribution centred on 2.3 nm is observed for the Ti_sph_ as-synthesized powder (Figure 4b); it has a narrow shape which confirms that the mesoporous microspheres are formed by densely packed primary nanoparticles with uniform agglomeration. On heating, the narrow shape is preserved but with significant differences; while the sample heated on the MW oven keeps the bimodal distribution of pores centred on 2.7 nm (like in the as-synthesized powder), the sample conventionally heated has increased this value up to 4.3 nm, indicating that the pores have grown substantially in the electric furnace.

### Photocatalytic performance

**Figure 5:**
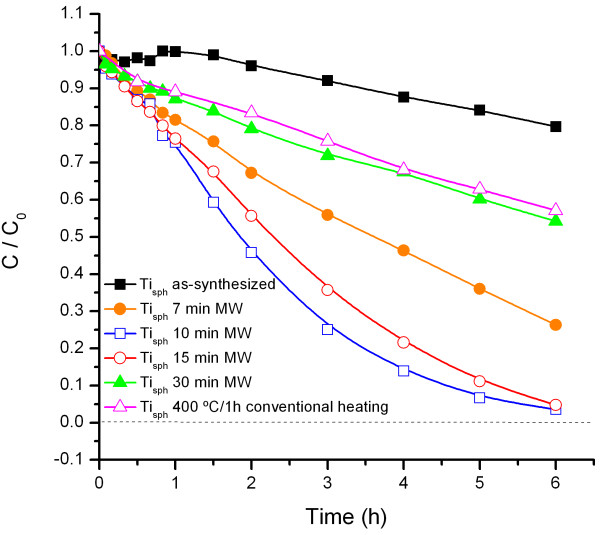
Evolution of methyl orange concentration during the photocatalytic test.

As described in the experimental section, the photocatalytic response of the obtained powders was estimated evaluating the degradation of methyl orange under UV-visible light. Figure 5 thus illustrates the decrease in the methyl orange concentration as a function of the reaction time for all those powders and, as observed, several interesting conclusions can be surmised. First, a thermal treatment of the TiO_2_ powder is by all means required. With the as-synthesized spheres, we attain the highest specific surface (Table 1), but merely a 10% to 20% of the starting methyl orange is degraded after the photocatalytic process, this certifying the importance of a certain degree of crystalline order for an effective catalysis. Second, the microwave heating that we propose here is clearly more efficient than the conventional electric heating typically used to improve the crystallinity of the particles. Actually, just a few minutes under the MW radiation are required to promote crystallization. Such a drastic reduction in the crystallization time allows the specific surface area and the porosity to retain high values, eventually leading to a better photocatalytic performance: as shown in Figure 5, when the as-synthesized TiO_2_ spheres are subjected to 10 to 15 min of MW sintering; the methyl orange is almost completely photodegraded after 6 h, this result being remotely accessible for a conventionally sintered powder.

## Conclusions

When conventional electric heating is applied to consolidate an amorphous powder of hierarchically nanostructured anatase microspheres, an increase in the crystal order is inescapably accompanied by a deleterious decrease in the specific surface and the porosity which dramatically reduces the photoactivity of this TiO_2_-based material. To avoid this scenario, microwave sintering has been successfully applied as an eco-friendly (energy saving) consolidation alternative: by reducing the heating time to just a few minutes, microwave radiation promotes the fast crystallization of the nanostructured microspheres, allowing the starting anatase powder to achieve a high crystallinity while keeping a high specific surface area and low density. As a straight consequence, the hunting of photons, the absorption of guest species and the photo-induced charge separation is fostered, eventually harvesting an improved photocatalytic performance.

## Competing interests

The authors declare that they have no competing interests.

## Authors’ contributions

DGC and TJ carried out the synthesis, crystallization methodology and photocatalytic studies, participated in the morphological characterization and drafted the manuscript. MP carried out the microscopy characterization and helped to draft the manuscript. ACC and DFH conceived of the study and participated in its design and coordination. All authors read and approved the final manuscript.
